# Another Light on the Milk Problem

**Published:** 1914-02-28

**Authors:** 


### ANOTHER LIGHT ON THE MILK PROBLEM.

### The Keeping Properties of Condensed Milks.

The medical profession and all the health agencies which take an interest in infancy and infant rearing have long been reiterating the uncontestable supremacy of mother's milk over all other kinds of diet for young babies. The truth of this cannot be for a moment denied; and there is another article of faith for mothers which is equally well established—namely, that fresh cow's milk, free from impurities as far as may be, is the ideal foundation for an artificial diet.

Nevertheless, there are occasions, few in proportion to the total infant population, yet not in themselves despicable in point of numbers, when neither of these methods of feeding is possible. The impossibility may be temporary, as, for instance, on a sea voyage; or permanent, as, for instance, in a region where cow's milk is unprocurable at a price within the reach of slender purses.

Thus it comes about that no inconsiderable section of the British race feeds its young perforce upon diets which are derived from milk by artificial preparation, designed to secure keeping properties, freedom from impurities, particularly germs, and other aims. Some of these foods are intended to be desiccated milk, requiring only the addition of water to fit them for an infant's consumption. Others, and the more popular kinds at present, belong to the "condensed milk" genera. Of these latter there are two main varieties recognised in text-books of infant dietetics—those with added sugar and those without. One of the principal differences between these two classes, from the practical point of view, lies in the fact that condensed milks without added sugar rapidly "go bad" if kept for more than a short time unused after the tin is opened. Those with sugar added can be safely used for some few days, especially if the tin is opened by the "two holes" method and carefully corked between whiles.

Deterioration of condensed milks is naturally more likely to occur in warm weather or in hot climates.

There is, however, another form of deterioration to which condensed milk is sometimes, though not very often, liable, and that is decomposition in the tin before it is opened. Such an accident is more likely to occur in milk which has been a long time in store, and is also facilitated if the temperature at which the tins have been kept is high. Provided the contents of the tins are sterile when the latter are sealed, it may be said that this change is almost unknown in the unsweetened brands of condensed milk; it is those with added sugar which sometimes evince it.

The nature of the changes involved has been lately investigated by Lieut.-Colonel W. W. O. Beveridge, D.S.O., whose conclusions are published in the *Journal of the Royal Army Medical Corps*.* He finds that in certain brands of condensed sweetened milk a greater degree of solidity and a brown colouration are developed when the tins are kept at 98° F. for more than a month. The manufacturers are aware of this fact also; but as the brown colour diminishes when water is added, and there is no evident alteration in taste, they have not considered the change injurious to health.

Colonel Beveridge finds that this change occurs more readily when cane sugar is added to the milk than when other forms of sugar are used. Also that the change involves a small proportion of the proteins present, which are rendered insoluble, and appear as a curdy precipitate when water is added to the milk. He further finds that bacteria are always present in milk which undergoes this change; that is, that such milks are not really absolutely sterile. The reason for this would appear to be that sweetened condensed milk is sterilised at a temperature of 80° to 85° C. in its manufacture; whereas unsweetened kinds are heated to 110° to 113° C. before canning, and to 120° C. afterwards. The reason why sweetened milks are not so highly heated is that reliance is placed upon the added sugar to help in the process of sterilisation. "It is evident, therefore," as this author says, "that the heat applied to sweetened milk is not sufficient to ensure sterility, and that the sugar merely acts as an antiseptic in temperate climates."

He further observes that there appears to be no marked degradation change in the constituents of the milk; nor any marked alteration in their relative proportions, with the exception of a slight increase of the reducing sugars and acidity. The contents of a tin of milk which have become coloured in this way will be found to have a greater consistency, and to be less miscible with water.

The cause of the brown colour is the action of certain bacteria upon sugar, especially upon glucose. This action is favoured by the presence of any salt of iron. Since the coating of true tin on the inner wall of the ordinary "tin" is exceedingly thin, there is always the possibility of minute breaches on the surface, admitting of contact between the milk and the iron which forms the basis of the wall of the can. In support of this contention it has been noticed that in some cases the brown colouration is not evenly distributed through the milk, but is greater at the bottom and along the sides. Moreover, iron can be detected by analysis in the milk which has undergone this particular change. It is not suggested that the iron alone accounts for the brown colour, but merely that it favours and accentuates it.

The concluding recommendation of the report says: "There is no doubt that to obtain better value and to obviate the risk of such a change occurring, especially when milks have to be stored for a considerable period, only those brands of unsweetened milks which have been proved to be sterile should be selected." This is not, of course, to condemn sweetened milks in the ordinary British climate.

* January, 1914, pp. 1-8.

